# How to Effectively Communicate Dismal Diagnoses in Dermatology and Venereology: From Skin Cancers to Sexually Transmitted Infections

**DOI:** 10.3390/diagnostics15030236

**Published:** 2025-01-21

**Authors:** Giulia Ciccarese, Francesco Drago, Astrid Herzum, Mario Mastrolonardo, Laura Atzori, Caterina Foti, Anna Graziella Burroni

**Affiliations:** 1 Section of Dermatology, Department of Medical and Surgical Sciences, University of Foggia, Viale Pinto 1, 71122 Foggia, Italy; mario.mastrolonardo@unifg.it; 2 Casa di Cura Villa Montallegro, 16100 Genoa, Italy; francescodrago007@gmail.com; 3 Department of Medical Specialties, ASL 3 Genovese, Via Assarotti 35, 16122 Genoa, Italy; astridherzum@yahoo.it; 4 Dermatology Clinic, Department of Medical Sciences and Public Health, University of Cagliari, 09042 Cagliari, Italy; atzoril@unica.it; 5 Section of Dermatology and Venereology, Department of Precision and Regenerative Medicine and Ionian Area (DiMePRe-J), University of Bari “Aldo Moro”, 70121 Bari, Italy; caterina.foti@uniba.it; 6 Section of Dermatology, Department of Health Sciences (Di.S.Sal), University of Genoa, IRCCS-San Martino Polyclinic Hospital, 16122 Genoa, Italy; annagraziella.burroni@unige.it

**Keywords:** dismal diagnosis, communication in dermatology, difficult discussion, bad news, sexually transmitted infections

## Abstract

**Background/Objectives**: One of the problematic situations dermatologists face with their patients is communicating dismal diagnoses. Examples are the diagnosis and prognosis of skin cancers like melanoma and Merkel cell carcinoma and the disclosure of the chronic nature of a disease that requires long-term therapies or can lead to scarring or disfiguring conditions. Likewise, receiving a diagnosis of a sexually transmitted infection can be a shocking event that can also put into question the patient’s relationship with his/her partner/partners. Some oncology and internal medicine protocols have been developed to support delivering distressing information. Regrettably, no consensus guidelines exist in dermatology, sexually transmitted infections, or other medical specialties. **Methods**: The protocols available in the literature to guide the disclosure of a dismal diagnosis have been reviewed in the present work. **Results**: The different protocols consist of several steps, from 5 to 13, and most of them are summarized by acronyms, such as “SPIKES”, “ABCDE”, and “BREAKS”. The frameworks are listened to and explained in the manuscript. **Conclusions**: These communication models are suggested to be adapted to dermatology and sexually transmitted infections. Indeed, several studies demonstrated that training in communication skills and techniques to facilitate breaking bad news may improve patient satisfaction and physician comfort.

## 1. Introduction

Among the problematic situations dermatologists face with their patients is communicating a dismal diagnosis or bad news, defined as “any information which adversely and seriously affects an individual’s view for his/her future” [1]. This news in dermatology is mainly related to oncological diseases. Some examples are the diagnosis and prognosis of life-threatening skin cancers, such as cutaneous or mucosal melanoma, Merkel cell carcinoma and Kaposi sarcoma, evidence or recurrence of secondary malignant disease, or disclosure of the positive result of genetic testing investigating the presence of pathogenic variants in melanoma susceptibility genes [2,3,4].

Other difficult discussions for dermatologists concern the diagnosis of a scarring or disfiguring condition (such as vitiligo and cicatricial alopecia) and the disclosure of the chronic nature of a disease such as lupus erythematosus, pemphigus vulgaris, hidradenitis suppurativa, and others that require long-term treatment and lifestyle changes to enhance recovery [5,6].

Noteworthy, it is usually difficult to communicate the diagnosis of a sexually transmitted infection (STI), such as human immunodeficiency virus (HIV), syphilis, or human papillomavirus (HPV) infection. Indeed, these diseases often put into question the patient’s relationship with his/her partner/partners and are often associated with feelings of guilt and fear of transmitting the infection to others [6]. Despite advances in antiretroviral treatment and the widely diffuse message that failure to detect the virus is equivalent to non-transmissibility (undetectable = untransmittable, U = U), a very recent study on the experiences of people recently diagnosed with HIV found that diagnosis was still a shocking event requiring careful support. In addition, there was ongoing stigma, shame, and reduced sexual confidence following diagnosis, and all these issues negatively impacted the quality of life [6,7].

Physicians may find it challenging to communicate unfavorable diagnoses for several reasons: the fear of being blamed for the dismal disease and triggering an aggressive reaction in the patient, the concern about not knowing all the answers to the patient’s questions and the fear of revealing their emotional involvement; moreover, the personal dread of illness and death, which can make communication even more difficult [8,9].

Breaking bad news can be particularly stressful when the clinician is inexperienced, the patient is young, or the possibility of successful treatment is limited [10]. The communication process can be even more challenging when dealing with patients who suffer from psychiatric disorders. Picardi A. et al. found that the overall prevalence of psychiatric morbidity in all adults attending the outpatient clinics of a dermatological hospital in Rome was 25.2%. Surprisingly, higher prevalence rates of psychiatric disorders (>30%) were observed not among patients with skin cancers but among patients with acne, pruritus, urticaria, alopecia and herpesviruses infections and in subjects without objective signs of dermatological disease [11]. Communication with such patients may be more complicated for dermatologists because, in the context of a depressive, anxiety, or somatoform disorder, they may be afraid of exacerbating the patient’s depressive/anxious symptoms or because they may be incapable of empathizing with the patient.

In general, the diagnosis of cancer itself is associated with significant psychosocial morbidity: patients are worried about uncertainty on their future, express feelings of helplessness or a sense of failure; they may feel isolated and stigmatized and suffer from body image problems; one-third of them develop affective disorders (anxiety and depression) and exhibit sexual dysfunction [12]. Noteworthy similar psychological issues are common in dermatological patients suffering from chronic STIs [6], even if they often do not express such problems during medical consultations [12].

The way in which healthcare professionals communicate with patients has implications for the quality of the provider-patient relationship, the patient’s adjustment and satisfaction, and the professional’s satisfaction and well-being (less burnout) [12,13].

Several studies have shown that patients to whom bad news is presented unhurried and empathetic or who receive more detailed information about their diagnosis report greater satisfaction and less emotional distress [12]. A well-orchestrated physician–patient communication may influence the patient’s psychological outcome and have a positive therapeutic effect [14,15].

Several oncology and internal medicine protocols have been developed, primarily based on expert opinion, to support delivering distressing information [16,17,18,19]. Very few studies concern the communication techniques of unfavorable diagnoses in dermatology and STIs [5,20,21], one of which was published in a non-English language [5]. Therefore, no specific consensus guidelines suggest how to provide dismal diagnoses in dermatology and STIs. Accordingly, the protocols already used to communicate a cancer diagnosis by oncologists may be applied in other contexts, such as dermatology and STIs.

The frameworks available in the literature to guide the delivery of distressing (mainly cancer-related) information and difficult discussions between physicians and patients are listened to and explained below. These protocols consist of several steps, from 5 to 13, and most of them are summarized by acronyms, such as “SPIKES”, “ABCDE”, and “BREAKS”.

Below are summarized also the few studies and documents addressing the issue of communication with patients at risk for or affected by STIs. Although there are no validated step-by-step protocols to guide the communication of an STI diagnosis, some works suggest helpful actions in managing these difficult situations [21,22].

## 2. Relevant Sections

### 2.1. The Australian Guidelines for Breaking Bad News

A consensus panel of health professionals and cancer patients from Australia developed the first consensus guidelines for delivering a cancer diagnosis, which were published in 1995. These guidelines suggest 13 steps: (1) have a private and comfortable place; (2) ensure adequate time during the first consultation; (3) assess the patient’s understanding; (4) provide straightforward information and repeat it; (5) encourage the patient to express feelings; (6) respond to the patient’s feelings with empathy (touch the hand, for example); (7) give a broad time frame for prognosis; (8) give realistic treatment aims; (9) schedule a next consultation to review the situation; (10) discuss treatment options; (11) offer assistance in breaking bad news to others; (12) provide information about supportive organizations (for example, cancer patient association); (13) write the information in a document that can be sent to other specialists [16].

### 2.2. The SPIKES Protocol

Five oncologists from Canada (Toronto Sunnybrook Regional Cancer Centre, Toronto) and the USA (University of Texas, MD Anderson Cancer Center, Houston, Texas) developed the SPIKES method. This method was first presented in 1998 at a meeting of the American Society of Clinical Oncology (ASCO); their paper was published in 2000. The five doctors’ main idea was “Before you tell, ask”. By creating a comfortable environment and obtaining the patient’s permission, physicians can help patients better control the information they want to know [17]. The method is composed of six steps.

The first consists of providing an adequate “Setting” (S): Ensuring privacy in a room, avoiding interruptions, making the patient sit down, and possibly removing barriers such as an office desk; if a room is not available and the patient is hospitalized, drawing the curtains around the patient’s bed; have tissues ready in case the patient becomes upset; maintaining eye contact to establish rapport, touching the patient on the arm or holding a patient hand if it is comfortable for the patient, and involving one or two family members chosen by the patient in the consultation.

The second step involves investigating the patient’s “Perception” (P) of the medical situation. This perception refers to the patient’s level of knowledge about the clinical problem and thoughts about this status. Based on this perception, the physician can correct misinformation and tailor the bad news to the patient’s understanding. Before discussing the medical conditions, the clinician can use open questions to create a picture of how the patient perceives his/her disease. For example, “What have you been told about your medical situation so far?” or “What is your understanding of the reasons we did the laboratory investigations/the magnetic resonance imaging”?

Step 3 consists of obtaining the patient’s “Invitation” (I) to disclose more or less information about the disease: assess how much detail the patient would prefer to receive regarding diagnosis, prognosis, and treatment. Examples of questions would be, “How would you like me to give the information about the laboratory/instrumental test results? Would you like me to give you all the information regarding the disease or only sketch out the results and spend more time discussing the treatment plan?”

Step 4 focuses on “Knowledge” (K): The patient should be prepared for the bad news with sentences like “Unfortunately, I have got some bad news to tell you”; bad news should be given using non-technical words, such as “sample of tissue” instead of “biopsy”, “spread” instead of “metastasized” and should be provided in small pieces, checking the patient’s understanding periodically; the following medical/surgical steps/options should be explained, and all the essential information should be written.

Step 5 consists of “Empathy” (E): This means to recognize the emotional reactions of the patients varying from silence to disbelief, crying, denial or anger; after observing for any emotion, ask the patient what he/she is thinking and demonstrate understanding of this feeling. Examples of an empathic response to acknowledge the patient’s emotion can be “I also wish the news were better”; this sentence can be followed by a validating statement to legitimate the patient’s feeling, for example, “I can understand how you feel”.

The last step (6) is to create a “Strategy and Summary”: Check the patient’s understanding of the discussion; repeat the treatment options and the following medical steps; establish a follow-up visit; invite questions and offer information about psychosocial support, such as the presence of patient support groups/associations [17].

The steps of the SPIKES protocol have been summarized in Table 1.

Approximately 13 years after its publication, a group of German oncologists and psychologists showed that some adjunctions to the SPIKES protocol could increase the patient’s perception and satisfaction of the first cancer disclosure: a frequent reassurance of the patient’s understanding, the perpetual possibility to ask question, respect for prearrangement needs and the conception of bad-news delivery in a two-step procedure: a first consultation and a second follow-up visit [23].

### 2.3. The ABCDE Protocol

Developed by Dr. Rabow and Dr. McPhee (Division of Internal Medicine, University of California, USA) and published in 1999, this protocol is summarized by the simple and mnemonic acronym “ABCDE” [24]. Subsequently, this model has been modified with additional material from other sources [18].

“A” stands for “ADVANCE PREPARATION”: Plan adequate time in a private location, ensure no interruptions, review the patient’s clinical history, and prepare yourself emotionally.

“B” stands for “BUILD A THERAPEUTIC RELATIONSHIP”: Identify the patient’s willingness to have more or less detailed information; ask the patient to choose a supportive person; anticipate bad news with sentences like “I am sorry, but I have bad news”. Assure the patient of your availability in the future.

“C” means “COMMUNICATE WELL”: Ask what the patient or his/her family already knows about the disease; be direct but compassionate in the disclosure of the diagnosis: do not use euphemisms, but use the words “cancer” and “death”; allow the time for silence and tears; ask the patient to tell you his/her understanding of what you have communicated; repeat the essential information and write them down.

“D” stands for “DEAL WITH PATIENT AND FAMILY REACTIONS”: Empathically respond to the patient and family’s emotional reactions. It could be appropriate to say, “I am sorry”.

“E” means “ENCOURAGE AND VALIDATE EMOTIONS”. Even when resolutive therapy is not available, offer hope and encouragement about the available options to alleviate the symptoms; consider an interdisciplinary approach to improve patient care (for example, hospice, psychology service support); inquire about the patient’s resources, asking, for example, “When bad things happened to you before—how have you coped?” [18,24].

The steps of the ABCDE model have been summarized in Table 2.

### 2.4. The BREAKS Protocol

The BREAKS model was developed in India by three doctors in the Departments of Oncology, Palliative Medicine, and Anesthesiology and published in 2010 [19]. The acronym ‘BREAKS’ summarizes the protocol’s six steps.

The “background” (“B”) is the first aspect to consider: In addition to studying the patient’s disease status, the physician should investigate the patient’s emotional status, cultural background, social context and familiar support system.

The “R” means “rapport”: Establish a good rapport with the patient without a mastery attitude. Avoid hurried manners.

“Exploring” (“E”) what the patient already knows and thinks about his/her illness can be helpful for the physician. Indeed, when attempting to communicate a dismal diagnosis, it is easier to start from what the patient already knows and possibly confirm the bad news rather than break it. Conflicts between the patient’s beliefs and actual conditions can be identified.

The “A” stands for “announce”. Bad news should be announced after the patient gives consent; indeed, the patient can decide to know the diagnosis or to be unaware of it. Information should be provided, avoiding technical terms.

“Kindling” (“K”) means to kindle the patient’s emotions. The patient can respond to the diagnosis with silence, tears, denial, or gallows humor. Ensure that he/she understands the realistic course of the illness with or without therapeutic options.

The last step consists of “summarizing” (“S”) the consultation with a few key points. A written summary is advisable, offering availability for the future and disclosing information to others. Even maintaining an optimistic attitude, the patient may feel highly desperate and may try to commit suicide; therefore, make sure about his/her safety once he/she leaves the room. For example, it should not allow the patient to drive back home alone.

The steps of the BREAKS protocol have been summarized in Table 3.

### 2.5. Counseling in the Field of STIs

A recent systematic review conducted by a group of oral medicine practitioners from Brazil and the USA attempted to collect studies regarding the communication of positive STI results [21]. They found four “protocols” and 18 observational studies. The four “protocols” cited in this review refer to the guidelines of the World Health Organization (WHO) [25], Center for Disease Control (CDC) [26], and the Brazilian Government for preventing and treating STIs [27,28], all published between 2005 and 2022. The first two guidelines were in English [25,26], and the others were in Portuguese [27,28]. However, unlike the previously mentioned protocols for disclosing dismal diagnoses (SPIKES, ABCDE, BREAKS), these four documents did not provide sequential and ordered steps to guide the communication of an STI diagnosis. They focused on the education and counseling of persons at risk of STIs, of persons who are infected with an STI and of their partners.

#### 2.5.1. WHO Essential Practice Guidelines in the Field of STIs

The WHO guidelines recommend the following actions for providing education and counseling to a patient with suspected or confirmed STIs:

- Welcome the patient warmly by name and introduce oneself;
- Ensure that privacy will be respected and sit close enough to talk comfortably and privately;
- Make eye contact and look at the patient as he/she speaks; use language that the patient can understand;
- Try to understand the feelings, experiences, and points of view of the patient;
- Encourage the patient’s conversation with signs of agreement (nod) and open-ended questions (for example, “Do you have any questions or concerns about your sexual health?”);
- Provide relevant information: STI transmission, symptoms, consequences, treatment and prevention; benefit of a healthy sexual life; when and how to seek advice about problems;
- Identify the patient’s concerns;
- Suggest several options (for prevention and, when available, for treatment);
- Be respectful of the patient’s choices;
- Check that the patient has understood what they have been told by having them repeat the crucial information;
- Do not keep moving in and out of the room; do not express judgmental comments [25].

The counseling contemplates a flexible approach: messages should be adapted to be relevant for each person. Counseling young people like adolescents may take longer times and requires some special considerations that have been listed below:

- Young people must feel confident that their privacy will be respected;
- Be sensitive to the possibility of sexual violence or coercion: Having sex with much older partners may be more likely to be forced and may carry a higher risk of STIs;
- Make sure the young person understands normal sexual development, how pregnancy occurs and that it is possible to say “no” to sex;
- Discuss issues related to substances of abuse, alcohol intake and sexual risk [29];
- If possible, involve peers in sexual education;
- Check that the adolescent can provide the drugs necessary to treat an STI and that he/she will be able to take the entire course of treatment;
- Establish a follow-up visit [25].

#### 2.5.2. CDC Guidelines on STI Treatment

These guidelines focused entirely on STI treatment and prevention. The authors provided some hints about good physician–patient communication: an effective interview and counseling should be characterized by respect, compassion, and a nonjudgmental attitude toward all patients. These skills are considered essential to obtaining a complete sexual history, which is the first step in identifying vulnerability and providing tailored counseling and care [26].

The Five Ps approach for obtaining sexual histories is based on open questions investigating the Partners, Practices, Protection from STI, Past history of STI, and Pregnancy intention:

1. Partners: “What is the gender(s) of your partner(s)?”
2. Practices: “To understand any risks for STIs, I need to ask specific questions about the kind of sex you have had recently”.
3. Protection from STIs: “Do you and your partner(s) discuss prevention of STIs?”
4. Past history of STIs: “Have you ever been diagnosed with an STI?”
5. Pregnancy intention: “How important is it to you to prevent pregnancy?” [26].

Supplementary Table S1 summarizes the key messages indicated by the STI guidelines on the patient’s counseling.

#### 2.5.3. Suggestions for an Effective Communication of STI Diagnosis

Kirschnick et al. recently published a systematic review of observational studies investigating physicians’ experiences when disclosing an STI diagnosis and patients’ experiences in receiving an STI diagnosis (mainly HIV infection). The authors found that communicating a positive STI test affects patients in several aspects, such as treatment adherence and relationship with the community. The importance of conducting personal and private discussions with patients to address their concerns and answer their questions has been highlighted. Regrettably, most studies indicated that post-test counseling was ineffective, and many physicians/nurses lacked preparedness for these difficult conversations, leading to suboptimal communication and potentially negative consequences for patients [21].

To fill the main gaps in this field, the authors proposed a key-points process that clinicians should follow to communicate an STI diagnosis:

- Study the features of the disease;
- Do not express personal feelings and opinions during the conversation;
- Establish a trusting relationship;
- Ensure privacy and confidentiality;
- Disclosure information; Discuss risks and preventive strategies;
- Establish a treatment plan and a follow-up visit;
- Address the patient’s questions and fears;
- Deal with patient’s and other people’s reactions;
- Encourage partner’s notification and treatment [21].

Another non-systematic review of the disclosure of HIV-positive results highlights several challenging situations that physicians have to encounter [22]. Physicians should be aware that when facing HIV positivity, the patients experience remarkable stress and a transition into three difficult periods: first, when the diagnosis is established, which is a significant psychological trauma with suicidal risk; the second period corresponds to the appearance of the clinical signs of the disease with the development of opportunistic infections in patients who have poor adherence to the antiretroviral treatment; the third critical period coincides with the terminal phase of the disease [22]. The introduction of Highly Active Antiretroviral Therapy (HAART) has greatly improved the quality of life, prognosis and acceptance of the diagnosis. However, a variable proportion of patients have poor adherence to HAART, and this is the main reason for treatment failures and HIV-related complications.

Moreover, the impact of the virus on the central nervous system (CNS) provides additional challenges in physician–patient communication. HIV enters the CNS early in the course of the infection and can cause severe conditions, such as HIV-associated progressive encephalopathy and AIDS dementia complex. As a consequence, patients with cognitive difficulties have further problems with compliance and adherence to their medication regimen.

The authors of this review concluded that conducting an effective physician–patient consultation is a complex skill that is gradually learned and perfected during a medical career. Good communication in HIV disease requires professional competence, good communication skills, ethical behavior, respect for the patient’s dignity, good teamwork skills, and confidentiality [22].

## 3. Discussion

Although clinicians are responsible for communicating dismal diagnoses, this skill is rarely taught in medical schools or residencies other than oncology, and clinicians need more training. Oncologists and oncology residents taught the SPIKES protocol reported an increased ability to disclose unfavorable medical information to patients [17]. Indeed, the SPIKES protocol, the most popular model proposed to deliver bad news, has reached guideline status in several countries and is used for training communication skills in the context of bad news announcements [23]; regrettably, this only applies to oncology medical practice.

The SPIKES model has several features in common with the other described frameworks. An adequate private setting and time for the medical consultation are always recommended. Interestingly, all the protocols advise switching off the telephone and the beeper when setting up the consultation to avoid interruptions [16,17,18,19,24]. In all the step-by-step models, before communicating the bad news, the physician should investigate what the patient already knows about the disease to identify misinformation that needs correction or to have a starting point for delivering the bad news. The unfavorable diagnosis should be disclosed after asking the patient the level of detail of information he/she wants to have, and the patient should be warned that bad news is coming. All the protocols agree on the importance of using easy sentences, avoiding technical words, repeating the critical information, and writing them down.

During the knowledge step, the SPIKES protocol suggests avoiding excessive bluntness (for example, sentences like “you have a bad cancer”) because the patient may feel isolated and angry [17]. However, the ABCDE protocol invites the physician to be direct using the words “cancer” and “death” [18,24]. Notable, in a study investigating the association between communication practices and patients’ psychological response, using the word “cancer” and discussing the severity of the situation are linked with lower levels of patient depression [15].

Also, during the knowledge step, the ABCDE model suggests avoiding euphemisms [18,24], whereas in the BREAKS model, euphemisms are welcomed so that the news will not explode like a bomb [19].

After providing information on the disease, time should be reserved to respond to the patient’s emotions. Every protocol validates the usefulness of summarizing the consultation and planning a follow-up visit [16,17,18,19,24].

Only the SPIKES protocol reserves a specific step for empathy [17], defined as ‘intuitively sensing the feelings of others, emotionally participating in the subjective states of others’ [30]. Neuroscience research has identified the mirror neuron system (MNS) as a potentially crucial neural substrate for empathy. Mirror neurons, sited in the prefrontal cortex and the insula of the brain, develop during infancy and childhood and are lacking when there are relational problems like autism [31]. Their key feature is that they modulate their activity when an individual executes a specific motor act and when they observe the same/similar act performed by another individual. Therefore, mirror neurons facilitate our learning by enabling us to understand and imitate the actions and behavior of those we observe [31]. A recent systematic review provides an overview of the empirical studies investigating the relationship between putative MNS activity and empathy in healthy populations.

In this work, empathy is considered a process consisting of three independent but interactive components: motor empathy (the automatic imitation of expressive body language, such as facial expressions, during the observation of another person), emotional empathy (the ability to resonate with the emotional state of another person), and cognitive empathy (the ability to comprehend the thoughts, feelings, beliefs and intentions of others; the ability to put oneself into another’s shoes) [32]. The analysis shows that emotional and cognitive empathy moderately correlates with MNS activity. However, these domains varied across techniques for acquiring MNS activity (transcranial magnetic stimulation, electroencephalogram, and functional magnetic resonance imaging) [32]. Interestingly, there is a gender difference in recruiting mirror neurons, with women seeming to recruit areas containing mirror neurons to a higher degree than males in empathic face-to-face interactions [4,33]. The importance of empathy in physician–doctor communication has been widely demonstrated. Patients who receive bad news in an unhurried and empathetic manner report greater satisfaction, less emotional distress and better overall adjustment to cancer [12]. To express empathy, physicians can use sentences that give the patient the feeling of being understood, for example: “I understand you’re suffering”, “I can imagine what you are going through”, “I can see things from your perspective”, and “This must be difficult for you.” [4].

The ABCDE protocol introduced the concept of compassion [18], which is different from empathy. Empathy is considered the least physicians can feel when a patient shares her/his feelings with them. Compassion means feeling other people’s suffering in a way that is very similar to pity but lacks the condescension that pity implies. With compassion, there is a deep communication between two human beings at the level of their humanity [4,18].

Unfortunately, there are no validated and ordered protocols for communicating a dismal diagnosis in the field of STIs. The existing guidelines, observational studies, and reviews mainly deal with general counseling pre- and post-STI testing and highlight some essential topics that need attention during communication [21].

Overall, the previously described methods provide a guide for communicating severe or disabling pathologies, but they are not yet entirely satisfactory for their purpose.

Several systematic reviews investigating different approaches for communicating dismal diagnoses (such as cancer) conclude that, due to the diversity among patients in terms of their needs and preferences, it cannot be concluded that any one method of communicating information accomplishes the stated goal or is superior to others [34].

In addition to physicians, nurses will increasingly be involved in situations where the information provided can impact patients’ lives and expectations. Consultation models that facilitate good communication, care, and compassion are also available for nurses. Indeed, nurses have an essential role in providing high-quality care. Developing a good relationship between patient and nurse is critical for engaging with service users and promoting a person-centered approach in several medical contexts [35]. Pendleton et al. (1984) proposed seven tasks that, together, taken in any sequence, form a coherent and comprehensive basis for any consultation. These tasks include paying attention to the patient’s needs, the doctor’s aims, and the shared desired outcomes. This model would allow the patient to feel part of a collaborative process and help build a solid relationship between physician/nurse and patient [36]. We believe a nurse should be involved early in the physician–patient consultation, as many patients may feel tension when they face a doctor. In contrast, they feel less stressed when talking and listening to a nurse.

A recent study on evidence-based methods for effective communication between nurses and patients with mental health issues explains how nurses can communicate compassionately, demonstrate cultural sensitivity, and develop trust within a therapeutic relationship, enabling them to effectively explore patients’ thoughts, feelings, and needs [37].

In addition to physicians and nurses, other trained counselors, such as psychologists and midwives (possibly gender-matched), might enrich the service of STI counseling and that of patients affected by skin cancer [26,38,39].

## 4. Conclusions

Although originating in oncology and internal medicine, the standardized protocols described in this work can be applied to disclosing unfavorable diagnoses in many difficult situations occurring in other medical specialties, such as dermatology and STIs. Indeed, the problematic discussions that dermatologists may have to face in their clinical practice are multiple and deal not only with oncological issues, such as cutaneous and mucosal melanoma but also with inflammatory and infective conditions: paraneoplastic skin diseases, such as pemphigus, dermatomyositis, and acrokeratosis paraneoplastic [5,40], inherited conditions predisposing to cutaneous and extracutaneous cancers [2,3,41,42], sexually transmitted infections that can be life-long and associated with cancers and infertility (genital or anal high-risk human papillomavirus infection, *Chlamydia trachomatis*, *Neisseria gonorrhoeae*, and some Mycoplasmas genital infections in women) [7,43,44,45], and infectious skin eruptions during pregnancy that can represent life-threatening health conditions for the fetus [46,47].

A recent French study showed the utility of simulation when disclosing melanoma diagnosis for dermatology residents. The advantages of simulation are based on the concept of experiential memories, which is an individual’s capacity to recall his/her experience of an event, even simulated, and his/her ability to make conscious use of the stored information when a similar event is experienced in clinical practice [48].

## 5. Future Directions

We know that the daily routine in a busy dermatological practice/service may not always allow all the steps of the proposed protocols to be fulfilled. However, they represent valuable advice for dealing with the most challenging situations.

We believe that applying frameworks such as SPIKES, ABCDE, and BREAKS to guide the disclosure of dismal diagnoses in dermatology and STIs may help physicians reduce stress and feel more comfortable in the complex situations of their clinical practice, as demonstrated in several studies [49,50,51].

Physicians’ acquisition of effective communication skills benefits patients as well, allowing them to better adjust to the disease [12,22]. Conversely, patients who were unhappy about how their diagnosis was communicated (mainly due to a lack of empathy from the doctor) were more likely to demonstrate long-term maladjustment [12,52].

Lastly, learning and improving communication skills when disclosing dismal diagnoses should be part of the training of every medical undergraduate and residency program. All university study courses in the healthcare profession (nurses, midwifery, and others) should provide training in communication skills.

## Figures and Tables

**Table 1:** Configuration of the SPIKES protocol.

Steps of the SPIKES Protocol	
S	Setting up interview
P	Assessing patient’s perception
I	Obtaining the patient’s invitation
K	Giving knowledge and information to patient
E	Addressing the patient’s emotions with an empathetic response
S	Strategy and summary

**Table 2:** Configuration of the ABCDE protocol.

Steps of the ABCDE Model	
A	Advance preparation
B	Build a therapeutic environment and relationship with the patient
C	Communicate well
D	Deal with patient and family reactions
E	Encourage emotions

**Table 3:** Configuration of the BREAKS protocol.

Steps of the BREAKS Protocol	
B	Background
R	Rapport
E	Explore
A	Announce
K	Kindling
S	Summarize

## Data Availability

This study did not create or analyze new data, and data sharing is not applicable to this article.

## Supplementary Materials

The following supporting information can be downloaded at: https://www.mdpi.com/article/10.3390/diagnostics15030236/s1, Table S1: Recommended actions in approaching STIs patient.

## References

[1] Buckman R. (1992). Breaking Bad News: A Guide for Health Care Professionals.

[2] Pastorino L., Andreotti V., Dalmasso B., Vanni I., Ciccarese G., Mandalà M., Spadola G., Pizzichetta M.A., Ponti G., Tibiletti M.G. (2020). Insights into Genetic Susceptibility to Melanoma by Gene Panel Testing: Potential Pathogenic Variants in ACD, ATM, BAP1, and POT1. Cancers.

[3] Ciccarese G., Dalmasso B., Bruno W., Queirolo P., Pastorino L., Andreotti V., Spagnolo F., Tanda E., Ponti G., Massone C. (2020). Clinical, pathological and dermoscopic phenotype of MITF p.E318K carrier cutaneous melanoma patients. J. Transl. Med..

[4] Poot F. (2009). Doctor-patient relations in dermatology: Obligations and rights for a mutual satisfaction. J. Eur. Acad. Dermatol. Venereol..

[5] Misery L., Legoupil D., Schollhammer M., Brenaut E., Abasq C., Roguedas-Contios A.M., Chastaing M. (2014). Annonce d’une mauvaise nouvelle en dermatologie [The announcement of bad news in dermatology]. Ann. Dermatol. Venereol..

[6] Ciccarese G., Drago F., Copello F., Bodini G., Rebora A., Parodi A. (2021). Study on the impact of sexually transmitted infections on Quality of Life, mood and sexual function. Ital. J. Dermatol. Venerol..

[7] Healey L.M., Markham S.R., Templeton D.J., Rabie L., Smith A.K.J. (2024). Stigma, support, and messaging for people recently diagnosed with HIV: A qualitative study. Sex. Health.

[8] Derry H.M., Epstein A.S., Lichtenthal W.G., Prigerson H.G. (2019). Emotions in the room: Common emotional reactions to discussions of poor prognosis and tools to address them. Expert Rev. Anticancer Ther..

[9] Buckman R. (1984). Breaking bad news: Why is it still so difficult?. Br. Med. J. (Clin. Res. Ed.).

[10] Ptacek J.T., Eberhardt T.L. (1996). Breaking bad news. A review of the literature. JAMA.

[11] Picardi A., Abeni D., Melchi C.F., Puddu P., Pasquini P. (2000). Psychiatric morbidity in dermatological outpatients: An issue to be recognized. Br. J. Dermatol..

[12] Ellis P.M., Tattersall M.H. (1999). How should doctors communicate the diagnosis of cancer to patients?. Ann. Med..

[13] Delvaux N., Razavi D., Marchal S., Brédart A., Farvacques C., Slachmuylder J.L. (2004). Effects of a 105 hours psychological training program on attitudes, communication skills and occupational stress in oncology: A randomised study. Br. J. Cancer.

[14] Sardell A.N., Trierweiler S.J. (1993). Disclosing the cancer diagnosis. Procedures that influence patient hopefulness. Cancer.

[15] Schofield P.E., Butow P.N., Thompson J.F., Tattersall M.H., Beeney L.J., Dunn S.M. (2003). Psychological responses of patients receiving a diagnosis of cancer. Ann. Oncol..

[16] Girgis A., Sanson-Fisher R.W. (1995). Breaking bad news: Consensus guidelines for medical practitioners. J. Clin. Oncol..

[17] Baile W.F., Buckman R., Lenzi R., Glober G., Beale E.A., Kudelka A.P. (2000). SPIKES-A six-step protocol for delivering bad news: Application to the patient with cancer. Oncologist.

[18] VandeKieft G.K. (2001). Breaking bad news. Am. Fam. Physician.

[19] Narayanan V., Bista B., Koshy C. (2010). ‘BREAKS’ Protocol for Breaking Bad News. Indian J. Palliat. Care.

[20] Cooke N., Colver G.B. (2016). How to convey a diagnosis of skin cancer: Patient preferences. Br. J. Dermatol..

[21] Kirschnick L.B., Calderipe C.B., Villa A., Santana Dos Santos E., Migliorati C., Martins M.D., Santos-Silva A.R. (2024). Patient communication protocols for sexually transmitted infections: A systematic review. Spec. Care Dentist..

[22] Tzaneva V., Iacob T. (2013). A better communcation with the patients improves the management of HIV disease: A nonsystematic review. Clujul. Med..

[23] Seifart C., Hofmann M., Bär T., Riera Knorrenschild J., Seifart U., Rief W. (2014). Breaking bad news-what patients want and what they get: Evaluating the SPIKES protocol in Germany. Ann. Oncol..

[24] Rabow M.W., McPhee S.J. (1999). Beyond breaking bad news: How to help patients who suffer. West. J. Med..

[25] World Health Organization (2005). Sexually Transmitted and Other Reproductive Tract Infections. A Guide to Essential Practice.

[26] Workowski K.A., Bachmann L.H., Chan P.A., Johnston C.M., Muzny C.A., Park I., Reno H., Zenilman J.M., Bolan G.A. (2021). Sexually Transmitted Infections Treatment Guidelines, 2021. MMWR Recomm. Rep..

[27] Ministério da Saúde (2017). Diretrizes para Organização do CTA no Âmbito dA Prevenção Combinada e nas Redes de Atenção à Saúde/Ministério da Saúde, Secretaria de Vigilância em Saúde, Departamento de Vigilância, Prevenção e Controle das Infecções Sexualmente Transmissíveis, do HIV/Aids e das Hepatites Virais.

[28] Ministério da Saúde (2022). Protocolo Clínico e Diretrizes Terapêuti cas para Atenção Integral às Pessoas com Infecções Sexualmente Transmissíveis (IST)/Ministério da Saúde, Secretaria de Vigilân cia em Saúde, Departamento de Doençasde Condições Crônicase Infecções Sexualmente Transmissíveis.

[29] Ciccarese G., Drago F., Herzum A., Rebora A., Cogorno L., Zangrillo F., Parodi A. (2020). Knowledge of sexually transmitted infections and risky behaviors among undergraduate students in Tirana, Albania: Comparison with Italian students. J. Prev. Med. Hyg..

[30] Thinès G., Lempereur A. (1975). Dictionnaire GéNéRal des Sciences Humaines.

[31] Rizzolatti G., Craighero L. (2004). The mirror-neuron system. Annu. Rev. Neurosci..

[32] Bekkali S., Youssef G.J., Donaldson P.H., Albein-Urios N., Hyde C., Enticott P.G. (2021). Is the Putative Mirror Neuron System Associated with Empathy? A Systematic Review and Meta-Analysis. Neuropsychol. Rev..

[33] Schulte-Rüther M., Markowitsch H.J., Shah N.J., Fink G.R., Piefke M. (2008). Gender differences in brain networks supporting empathy. Neuroimage.

[34] Rodin G., Mackay J.A., Zimmermann C., Mayer C., Howell D., Katz M., Sussman J., Brouwers M. (2009). Clinician-patient communication: A systematic review. Support. Care Cancer.

[35] Buchanan P. (2017). Difficult discussions in dermatology: Communicating with compassion. Dermatol. Nurs..

[36] Harper C., Ajao A. (2010). Pendleton’s consultation model: Assessing a patient. Br. J. Community Nurs..

[37] Bond C. (2024). Skills for communicating effectively with people who have mental health issues. Nurs. Stand..

[38] Doyle A.M., Shaw L. (2002). Clinical Psychology Services in HIV and Sexual Health. A Guide for Commissioners of Clinical Psychology Services. The British Psychological Society Division of Clinical Psychology Briefing Paper No. 17. https://cms.bps.org.uk/sites/default/files/2022-07/briefing_paper_17_-_clinical_psychology_services_in_hiv_and_sexual_health.pdf.

[39] Peters E.M. (2012). Psychological support of skin cancer patients. Br. J. Dermatol..

[40] Gioe R., Mathien A., Kuraitis D. (2024). Acrokeratosis paraneoplastica (Basex syndrome) with trachyonychia preceding the diagnosis of squamous cell carcinoma of the lung. Dermatol. Online J..

[41] Ferrante L., Ortman C. (2024). Genetic principles related to neurocutaneous disorders. Semin. Pediatr. Neurol..

[42] Alchoueiry M., Lambert W.C., Henske E.P. (2024). Guiding the future of clinical care and clinical research in Birt-Hogg-Dubé. Eur. J. Hum. Genet..

[43] Drago F., Herzum A., Ciccarese G., Dezzana M., Casazza S., Pastorino A., Bandelloni R., Parodi A. (2016). Ureaplasma parvum as a possible enhancer agent of HPV-induced cervical intraepithelial neoplasia: Preliminary results. J. Med. Virol..

[44] Ciccarese G., Di Biagio A., Bruzzone B., Guadagno A., Taramasso L., Oddenino G., Brucci G., Labate L., De Pace V., Mastrolonardo M. (2023). Monkeypox outbreak in Genoa, Italy: Clinical, laboratory, histopathologic features, management, and outcome of the infected patients. J. Med. Virol..

[45] Shroff S. (2023). Infectious Vaginitis, Cervicitis, and Pelvic Inflammatory Disease. Med. Clin. N. Am..

[46] Rebora A., Ciccarese G., Herzum A., Parodi A., Drago F. (2020). Pityriasis rosea and other infectious eruptions during pregnancy: Possible life-threatening health conditions for the fetus. Clin. Dermatol..

[47] Pereira L. (2018). Congenital Viral Infection: Traversing the Uterine-Placental Interface. Annu. Rev. Virol..

[48] Dietrich E., Le Corre Y., Dupin N., Dréno B., Cartier I., Granry J.C., Martin L. (2021). Benefits of simulation using standardized patients for training dermatology residents in breaking bad news. Ann. Dermatol. Venereol..

[49] Mahendiran M., Yeung H., Rossi S., Khosravani H., Perri G.A. (2023). Evaluating the Effectiveness of the SPIKES Model to Break Bad News—A Systematic Review. Am. J. Hosp. Palliat. Care.

[50] Domar A.D., Korkidakis A., Bortoletto P., Gulrajani N., Khodakhah D., Rooney K.L., Gompers A., Hacker M.R., Grill E. (2024). The impact of an adapted SPIKES protocol vs routine care in the delivery of bad news to IVF patients: An exploratory pilot multicenter randomized controlled trial. J. Assist. Reprod. Genet..

[51] Zemlin C., Nourkami-Tutdibi N., Schwarz P., Wagenpfeil G., Goedicke-Fritz S. (2024). Teaching breaking bad news in a gyneco-oncological setting: A feasibility study implementing the SPIKES framework for undergraduate medical students. BMC Med. Educ..

[52] Omne-Pontén M., Holmberg L., Sjödén P.O. (1994). Psychosocial adjustment among women with breast cancer stages I and II: Six-year follow-up of consecutive patients. J. Clin. Oncol..

